# Optimisation of octinyl succinic anhydride starch stablised w_1_/o/w_2_ emulsions for oral destablisation of encapsulated salt and enhanced saltiness

**DOI:** 10.1016/j.foodhyd.2017.03.002

**Published:** 2017-08

**Authors:** Natalie Chiu, Amparo Tarrega, Christopher Parmenter, Louise Hewson, Bettina Wolf, Ian D. Fisk

**Affiliations:** a Division of Food Sciences, School of Biosciences, University of Nottingham, Sutton Bonington Campus, Loughborough, LE12 5RD, UK; b Institute of Agrochemisty and Food Science (IATA-CSIC) Avda, Agustin Escardino, 7, 46980, Paterna, Valencia, Spain; c Nottingham Nanoscale and Microscale Research Centre, University of Nottingham, University Park, Nottingham, NG7 2RD, UK

**Keywords:** OSA starch, Sodium encapsulation, Salt reduction, Emulsion, Control release, Sodium perception

## Abstract

Sodium (salt) was encapsulated within the inner water phase of w_1_/o/w_2_ food emulsions externally stabilised by starch particles with the ultimate aim of enhancing saltiness perception. The physical properties of the starch particles were modified by octenyl succinic anhydride (OSA) treatment (0–3%) to vary the degree of hydrophobicity of the emulsifying starch. During oral processing native salivary amylase hydrolysed the starch and destabilised the o/w emulsion releasing the inner w/o phase and subsequently sodium into the oral cavity, resulting in a salty taste. Whilst increasing OSA treatment levels increased the stability of the emulsion, intermediate or low levels of starch modification resulted in enhanced saltiness. It is therefore proposed that 1.5% OSA modified starch is optimal for sodium delivery and 2% OSA modified starch is optimal for sodium delivery in systems that require greater process stability. It is also shown that sodium release was further enhanced by oral processing and was positively correlated with native amylase activity. The results demonstrate a promising new approach for the reduction of salt or sugar in emulsion based foods.

## Highlights

• The ability of starch modified with OSA (0–3%) to stabilise w_1_/o/w_2_ emulsions was examined.
• Increasing OSA starch modification improved emulsion stability.
• Emulsions were perceived to be saltier with a less modified starch as a stabiliser.
• Intermediate OSA treatment levels are recommended for industrial use.
• Individual amylase activity affected time to maximum intensity of perception.

## Graphical abstract

**Figure:**
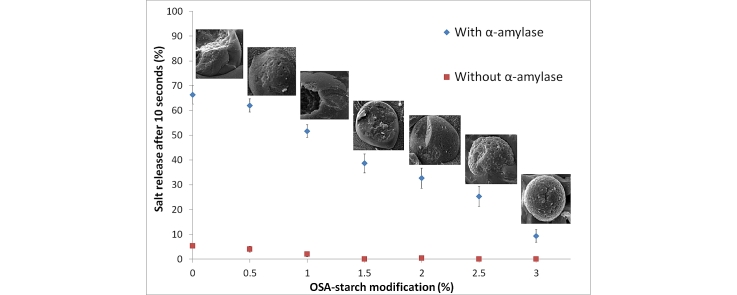

## 1 Introduction

Overconsumption of sodium remains an epidemic issue with salt as the main source of sodium in the human diet (Anderson et al., 2010). Although various salt reduction strategies have been explored for application in food products (He and MacGregor, 2008, Kilcast and Angus, 2007, Rama et al., 2013, Tian and Fisk, 2012), many are product or category specific and further efforts to reduce sodium consumption in food is needed, as the average global salt consumption remains above the recommended levels of 5 g/day (World Health Organization, 2012). The multifunctional role of salt increases the complexity of reducing sodium within the diet and furthermore reduction strategies must be delivered without compromising perception of the food product’s acceptability.

Complex emulsions (w_1_/o/w_2_) are present in many foods, these may be formed either by design or as an artefact of processing or transiently formed during mastication/cooking. Through careful design, complex water-in-oil-in-water (w_1_/o/w_2_) emulsions may be able to encapsulate free sodium within their internal water phase and release it in a targeted fashion during the short time period of oral processing. This may allow lower salt levels without compromising on saltiness perception. We have recently reported on the use of w_1_/o/w_2_ emulsions to reduce sodium levels in liquid or semi-liquid systems by modulating salt perception through targeted delivery of encapsulated salt in the oral cavity (Chiu, Hewson, Fisk, & Wolf, 2015). Commercial octinyl succinic anhydride (OSA) starch was used as an external emulsifier with two key roles: (i) Emulsion stabilisation prior to consumption entrapping internalised sodium within the inner water phase and (ii) emulsion destabilisation during oral processing which releases the entrapped sodium for perception. In a previous study, fat crystals were used to stabilise w_1_/o/w_2_ emulsions and based on the osmotic pressure sodium either remained in the w_1_ aqueous phase or moved to the w_2_ phase (Frasch-Melnik, Spyropoulos, & Norton, 2010).

OSA treatment results in the formation of regions of hydrophobicity on the surface of the starch particle, ultimately resulting in an amphiphilic starch particle, this modified starch is surface active and can act as a particle stabiliser for o/w and w_1_/o/w_2_ emulsions (Matos et al., 2013, Sweedman et al., 2013, Timgren et al., 2013, Yusoff and Murray, 2011). During oral processing, salivary amylase initiates starch digestion and during this initial process reduces the emulsification capability of OSA treated starch particles resulting in coalescence of the o/w emulsion and diffusion of the internal water phase into the saliva. This is in direct contrast to more stable complex emulsions, which do not destabilise in the oral cavity.

The present study furthers this concept, hypothesising that variation of the degree of OSA modification will modulate the rate of destabilisation (by coalescence) of the w/o/w emulsion during oral processing, and thus at lower levels of OSA treatment the inner salty water phase will be released more efficiently to elicit saltiness perception. Therefore, the aim of this study was to identify an optimum level of octinyl succinic anhydride (OSA) modification (within USA FDA permitted levels 0–3%) for waxy maize starch particles to maximise w_1_/o/w_2_ emulsion physical stability whilst still destabilising under oral processing conditions to release sodium for perception. Emulsion microstructure is monitored for 90 days to demonstrate shelf-life stability and sodium release was quantified *in-vitro* and *in-vivo* with accompanying sensory evaluation to determine saltiness perception.

## 2 Materials and methods

### 2.1 Materials

All materials used for starch modification and emulsion preparation were food grade. C*Gel 04201, a waxy maize starch containing approximately 95% amylopectin, was obtained from Cargill (Sas van Gent, Netherlands). Octenyl succinic anhydride (OSA) was donated by Vertellus (Pennsylvania, USA). Sodium hydroxide (NaOH) was obtained from VWR International Ltd. (Lutterworth, UK). Polyglycerol polyricinoleate (PGPR) used to stabilise the internal water phase (w_1_) was donated by Danisco (Dorset, UK). Following protocol in our previous publication (Chiu et al., 2015), samples designed to not be susceptible to oral breakdown were stabilised with pea protein isolate (PPI, Myprotein, Manchester, UK). Sunflower oil and table salt was purchased from a local supermarket. Calcium chloride (CaCl_2_), ethanol, hydrochloric acid (HCl), 4-morpholinepropanesulfonic acid sodium salt (MOPS sodium salt), phenolphthalein, porcine α-amylase, sodium azide and salivary amylase assay kit (MAK009) were obtained from Sigma-Aldrich (Gillingham, UK). Sodium azide was used as an antimicrobial agent and was only added to samples that were not intended for sensory analysis. Amyloglucosidase, d-glucose, standardised regular maize starch and thermostable α-amylase were provided as part of the Megazyme total starch assay kit (Megazyme, Co., Wicklow, Ireland). Deionised water, with a resistivity of 15 MΩ/cm was used for the preparation of all solutions.

### 2.2 Hydrophobic modification of waxy maize starch with octinyl succinic anhydride

C*Gel 04201 was hydrophobically modified by OSA treatment following Bhosale and Singhal (2006). C*Gel (125 g) was mixed with 475 mL deionised water using an overhead mixer with a four bladed propeller stirrer (EURO-ST D S2, IKA-WERKE, Staufen, Germany). The pH of the slurry was adjusted to pH 8.0 ± 0.2 by the addition of 2% NaOH solution. OSA up to 3% at 0.5% increments, based on the weight of starch, was added drop-wise to the slurry over a 2 h period at room temperature. During the addition of OSA, the pH was maintained at 8.0 ± 0.2 by the addition of 2% NaOH solution. The reaction was left to proceed for 24 h at 30 °C after which the pH was adjusted to 6.5 using 2% HCl. The slurry was then washed with water and centrifuged at 4193 g. This centrifugation process was repeated three times. The OSA starches were dried in an oven at 45 °C for 12 h and stored in a sealed container at room temperature until use. All steps of the modification procedure were repeated without the addition of OSA and the starch obtained from this process is considered as not hydrophobically modified. Albeit not corresponding to the original starch, C*Gel 04201 as obtained from the supplier, applied in this research, it is in the following referred to as unmodified starch.

### 2.3 Determination of the degree of substitution

The degree of substitution (DS) is the average number of hydroxyl groups substituted per glucose unit and was determined by alkali saponification and back titration of excess alkali with HCl according to Whistler and Paschall (1967). A suspension of the OSA starch (5 g of starch in 50 mL water) was mixed with 25 mL of a 0.5 M aqueous NaOH solution and stirred for 24 h. Excess alkali was titrated with 0.5 M HCl, using phenolphthalein as an indicator. A blank titration of a suspension of unmodified starch (5 g unmodified starch in 50 mL water) was performed and the difference in HCl added to the modified and unmodified starch suspension was assumed to be due to chemically bound OSA. OSA substitution (%) was then calculated with Equation (1).

(1) $$\%\;OSA=\frac{\left[\left(V_{Blank}-V_{Sample}\right)\times 0.1\times M\times 100\right]}{W}$$

where $V_{Blank}$ = volume of HCl required for back titration; $V_{Sample}$ = volume of HCl required for sample titration; M = Molarity of HCl; W = weight of sample taken (g).

DS was determined from % OSA substitution with Equation (2):

(2) $$DS=\frac{Mw_{g}\times \%\;OSA}{Mw_{OSA}\times 100-(\left(Mw_{OSA}-1\right)\times \%\;substitution)}$$

where $Mw_{g}$ = molecular weight of glucose residue (162); $Mw_{OSA}$ = molecular weight of *OSA* (210).

### 2.4 Emulsion preparation

To prepare the water-in-oil-in-water (w_1_/o/w_2_) emulsions (1 L) initially a water-in-oil emulsion (w_1_/o) containing 30 %w/w aqueous phase was formulated and then incorporated into the external water phase (w_2_) at a ratio of 1:1 to create a w_1_/o/w_2_ emulsion. A high shear overhead mixer (Silverson L5M fitted with emulsor screen, Chesham, UK) was used for all steps of emulsion processing. The internal water phase (w_1_) consisted of 141 mM aqueous NaCl solution and the oil phase contained 2.8% w/w PGPR 90 (premixed at 4000 rpm for 1 min and allowed to equilibrate). The aqueous phase was added to the oil phase and mixed for 2 min at 4000 rpm. The w_1_/o emulsion was subsequently mixed (at a ratio of 1:1) with w_2_ at 4000 rpm for 2 min. The external water phase consisted of a 4% w/w aqueous suspension of unmodified or OSA modified starch.

### 2.5 Microscopy, image analysis and droplet size

The droplets of the w_1_/o/w_2_ emulsions were visualised using optical microscopy and image analysis was applied to quantify the droplet size distribution and the surface based mean droplet diameter. Additionally, the method of cryo-Scanning Electron Microscopy (cryo-SEM) was applied to visualise the topography of the starch stabilised w/o/w droplet surfaces.

Optical micrographs were captured immediately and after 1, 3, 7, 30 and 90 days after the preparation of the w_1_/o/w_2_ emulsions using a digital inverted transmission light microscope (EVOS fl, Life Technologies Ltd., Paisley, UK) fitted with a 20× bright field, long working distance objective (AMEP4624, Life Technologies Ltd., Paisley, UK). A total of 600 droplets were measured for each sample using the image analysis software ImageJ (NIH, Bethesda, USA). The data for all formulations were analysed for the surface area based mean diameter (d_3,2_) and this parameter was used as characteristic diameter to indicate emulsion droplet size and microstructure stability for a period of up to 90 days. Whilst there are limitations to measuring absolute droplet size by microscopy, the data was used to compare across emulsions and to identify instability and was therefore appropriate.

Cryo-SEM was performed on a FEI Quanta 3D 200 dual beam Focused Ion Beam Scanning Electron Microscope (FIB-SEM) to evaluate the structure at the interface of the emulsions. Images were acquired using secondary electron imaging at an accelerating voltage of 5–15 kV.

### 2.6 Sodium measurements

The free sodium content of the w_1_/o/w_2_ emulsions was measured immediately and after 1, 3, 7, 30 and 90 days after emulsion formation using a sodium ion specific electrode with a measurement range of −1999.9 to +1999.9 mV (Jenway, Stone, UK). Sodium chloride (salt) solutions between 0 and 0.15 M were used to create a standard curve.

Sodium chloride release from the complex emulsions was quantified in vitro using methodology as previously adapted from Al-Rabadi, Gilbert, and Gidley (2009) in our recent publication (Chiu et al., 2015). Emulsion (10 mL) was mixed on a magnetic stirrer at 37 °C with 10 mL of 1 M aqueous carbonate buffer and porcine salivary α-amylase was added under continuous stirring. The final solution had an enzyme level of 50 units/mL. Measurements were recorded every second for 20 s to monitor the release of sodium from w_1_ to w_2_. After 20 s, 1 mL of 2 M HCl was added to the sample to inactivate the enzyme and 0.02% sodium azide was mixed into the sample to prevent microbial spoilage. The amount of released salt was expressed as the percentage of total salt using Equation (3).

(3) $$\%\;Released\;salt\;=\frac{m_{external}}{m_{total}}\times 100$$

where $m_{total}$ is the total mass of salt that was originally present in the internal phase and $m_{external}$ is the mass of salt that has moved to the external water phase.

### 2.7 Total starch assay

Following the measurement of sodium release, the undigested and digested emulsions were analysed for total starch to ascertain the degree of starch digestion. The same protocol was applied to *in-vivo* processed emulsions; the protocol for sample preparation is described in the sensory methodology section. A standard published protocol (AOAC Method 996.11, Megazyme International Ireland Ltd.) was followed which required the initial preparation of morpholinepropanesulfonic acid (MOPS) sodium salt and sodium acetate buffers. MOPS sodium salt buffer was prepared by dissolving 11.55 g of MOPS sodium salt in 900 mL of water, then adjusting to pH 7.0 by the addition of 1 M HCl dropwise. Calcium chloride (0.74 g) and 0.2 g of sodium azide was dissolved in the solution and the total volume adjusted to 1 L. The sodium acetate buffer was prepared with 11.6 mL of glacial acetic acid to 900 mL water adjusted to pH 4.5 by 1 M sodium hydroxide solution, 0.2 g of sodium azide was dissolved and the volume was adjusted to 1 L.

Samples (100 mg) were mixed with 5 mL of aqueous ethanol (80% v/v), and incubated at 80 °C for 5 min. An additional 5 mL of 80% v/v aqueous ethanol was added and the sample was then centrifuged for 10 min at 1800 g and the supernatant discarded. The pellet was re-suspended in 10 mL of 80% v/v aqueous ethanol, stirred on a vortex mixer, and centrifuged as previously described. The supernatant was poured off and immediately 2 mL of DMSO was added to the pellet and stirred on vortex mixer. The content was placed in a boiling water bath for 5 min. Thermostable α-amylase (3 mL) and 50 mM MOPS buffer (90 mL) was previously mixed and added to the heated content. The mixture was heated in boiling water for an additional 6 min. Sodium acetate buffer (4 mL) and 0.1 mL amyloglucosidase (20 U) were added to the samples followed by mixing and incubation at 50 °C for 30 min. The entire content was transferred to a 100 mL volumetric flask and the volume was adjusted to 100 mL using distilled water. An aliquot of the solution was centrifuged at 1800 g for 10 min. The concentration of glucose in the clear filtrate was then measured using a glucose analyser (Analox GM9 Analyser, London, UK).

### 2.8 Sensory testing

Overall saltiness perception and time-intensity data were acquired utilising the facilities of the Sensory Science Centre at the University of Nottingham. Prior to commencing the sensory studies full approval from the Ethics Committee at the University of Nottingham (G120222015 SoBS PhD) and signed informed consent from each volunteer was obtained.

For all studies, samples were evaluated within 1 day of sample preparation. The order of presentation of samples was randomised and balanced across the panel and samples were evaluated within 1 day of sample preparation and were labelled with three-digit codes.

#### 2.8.1 Overall perception of saltiness

Overall perception of saltiness from the emulsions was evaluated using a series of paired comparison tests (2-Alternate Forced Choice tests, BS ISO 5495:2007). 100 volunteers (58 females and 42 males) were recruited from the University of Nottingham and asked to attend one session. The volunteers were presented with a series of 10 pairs of emulsion samples. Samples (10 mL) were presented in odourless, plastic pots labelled with 3 digit codes; presentation order was randomised both within each pair and across the 10 paired comparison (PC) tests. For each PC, assessors were required to test the samples in the order presented and following a set test protocol; place the whole sample in the mouth, press the tongue against the palate three times, hold the sample for 10 s prior to swallowing, and then indicate which of the two samples they perceived to be saltier. Before and in-between testing samples, assessors were required to cleanse their palate through consumption of green apple slices (Granny Smith variety), unsalted crackers (99% Fat Free, Rakusen’s Leeds, UK) and mineral water (Evian, Danone, France). Within each PC, sample pairs had the same level of encapsulated salt but were stabilised with starches of different levels of OSA modification including 1.5, 2, 2.5 and 3% as well as the unmodified starch.

The test was used in forced-choice mode, so panellists were required to give an answer even if the perceived difference was negligible and panellists were given the opportunity to comment on the samples. Results were compared to BS EN ISO 5495:2007 to determine difference and similarity, respectively (British Standards Institution, 2007).

#### 2.8.2 Time-intensity and data analysis

Assessors (n = 10, 9 females, 1 male, aged 44–72 years) from the University of Nottingham external panel were recruited to take part. All had extensive experience of sensory evaluation using Time Intensity methods and in addition attended 2 h training sessions to familiarise them with the samples and calibrate the line scale used. Following the sampling protocol detailed in Section 2.8.1, panellists started rating their perception of saltiness immediately after the sample was placed in the mouth and continued rating for 30 s (swallowing the samples after 10 s according to the test protocol). The perceived intensity was recorded on a continuous line scale with data collected every 1s using the computerised data acquisition system, FIZZ 2.46 (Biosystems, Couternon, France). Each panellist attended a total of five 2 h sessions to evaluate all samples. A total of seven emulsion samples were evaluated (5 replicate assessments of each), each sample containing the same concentration of 0.141 M salt in the internal phase and stabilised with unmodified or OSA modified starch (0.5, 1, 1.5, 2, 2.5, 3% OSA).

Fizz Calculations normalisation and computation algorithm was used to extract key parameters from the time-intensity curves: *T*_max_ = time to maximum intensity, *I*_max_ = maximum intensity, Area under the curve = total perceived intensity and *I*_*10*_ = intensity at 10 s. All parameters were analysed with ANOVA followed by Tukey HSD post hoc test (IBM SPSS Version 22) to determine significant differences between the different emulsions (p < 0.05).

### 2.9 α-amylase activity and saliva flow rate

Individual’s saliva α-amylase activity was measured to understand the impact of salivary α-amylase concentration on sodium release in vivo and subsequent time to maximum saltiness perception. Nitrophenol standards between 0 and 20 nmole were produced using 2 mM nitrophenol and water for colorimetric detection. Quintuplicate samples of stimulated saliva were collected from the external trained panellists, as previously carried out. Panellists were instructed to expectorate saliva every 30 s for 5 min and the saliva collected was measured and the value was divided by the time the collection lasted to obtain salivary flow rate (mL/min). Saliva samples (100 mg) were centrifuged with 0.5 mL amylase assay buffer at 13,000 g for 10 min to remove insoluble material. Samples of 1 μL were transferred into the individual wells of a 96-well plate and 49 μL amylase assay buffer was then added. To each reaction, 100 μL master reaction mix, containing equal proportions of amylase assay buffer and amylase substrate mix, was added and mixed. The absorbance was measured in a microplate reader (LT-4000, Labtech, Sussex, UK) at 405 nm. The initial absorbance was measured after 2 min and the final absorbance was measured after 5 min. The background absorbance was corrected by subtracting the 0 nitrophenol standard from the measurement of the standards and samples. One unit of amylase is the amount of amylase that cleaves ethylidene-pNP-G7 to generate 1.0 μmole of *p*-nitrophenol per minute at 25 °C. The amount of nitrophenol generated between the two measured times were compared to the standard curve and the amylase activity was calculated using Equation (4):

(4) $$Amylase\;activity\;\left(milliunits\right)=\frac{C_{nitrophenol}\times dilution\;factor}{reaction\;time\;\times V}$$

where $C_{nitrophenol}$ is the difference of nitrophenol (nmole) between initial and final absorbance measured; dilutionfactor is 50; reactiontime is 5 min and V is the sample volume (mL) added.

The rate of amylase expression was calculated by dividing the amylase activity by the salivary flow rate.

## 3 Results and discussion

### 3.1 Modification of starch with octinyl succinic anhydride (OSA) treatment

**Fig. 1:**
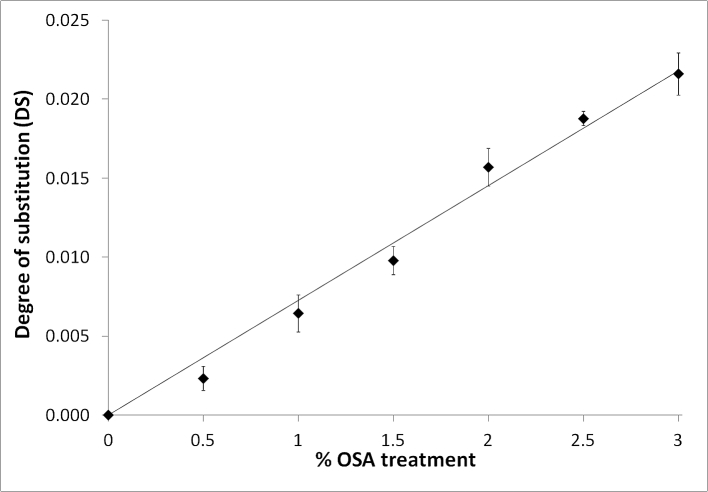
Degree of substitution of starch relative to concentration of OSA. Data are means of three replicates ± standard deviation.

The degree of substitution (DS) of starch after OSA modification was measured. Fig. 1 shows the DS for each OSA modified starch, starches were prepared as independent replicates. As the OSA concentration of the treatment solution increased the degree of substitution increased linearly (R^2^ = 0.98), suggesting the treatment was effective and that OSA treatment was not limited by surface area or reactant availability. The DS at the maximum treatment concentration of 3% OSA was 0.216 ± 0.014, which is in close agreement with previously reported results (Bhosale and Singhal, 2006, Liu et al., 2008).

### 3.2 Impact of OSA modification level (0–3%) on emulsion microstructure

**Fig. 2:**
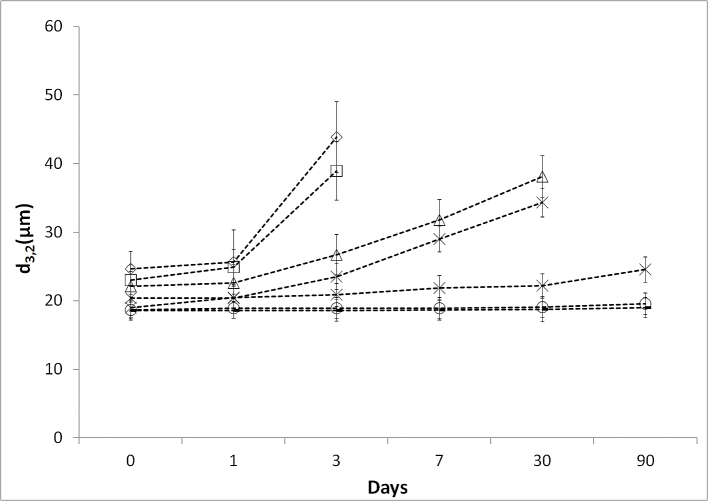
Mean droplet size (d3,2) of complex emulsions (4% w/w) over time prepared with 0% OSA modification (◇); 0.5% OSA-modification (□); 1.0% OSA-modification (△); 1.5% OSA-modification (✕); 2.0% OSA-modification (✱); 2.5% OSA-modification (+) and 3.0% OSA (ˆ). Emulsion droplet size measurements were ceased once coalescence of the more unstable emulsions was observed. Data are means of three replicates ± standard deviation.

**Fig. 3:**
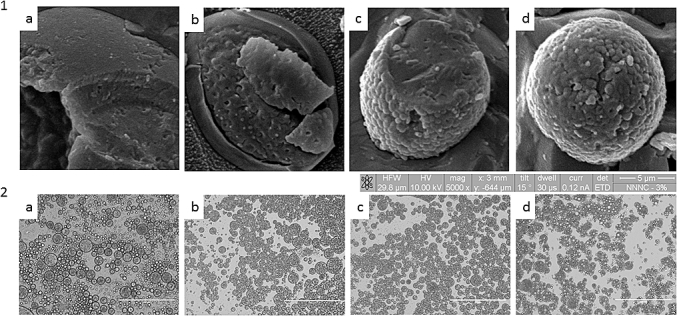
Cryo-SEM (1) and light microscopy (2) images of an oil droplet of the w_1_/o/w_2_ emulsion stabilised with unmodified - starch (a) and increasing level of OSA modification: 1.5% (b), 2% (c) and 3% (d). The scale bar in each light micrograph corresponds to 200 μm.

To evaluate emulsion stability, droplet size and emulsion microstructure were characterised by light microscopy with image analysis for droplet size (Fig. 2) and cryo-SEM for the evaluation of droplet surface topography (Fig. 3).

Increasing levels of OSA starch modification produced marginally smaller (p < 0.05) oil droplets (Fig. 2) and the emulsions with higher OSA treatment levels were more stable over 90 days storage time (p < 0.05). The initial droplet size of the emulsions, captured immediately after emulsification, ranged between 18 and 25 μm and was highest for the emulsion stabilised with the untreated starch and lowest for the emulsion stabilised with the 3% OSA starch. None of the emulsions showed a significant change in mean droplet size after 1 day of storage (p > 0.05), however, with increasing storage period, emulsion stability depended on the level of OSA modification. Emulsions stabilised with unmodified starch and 0.5% OSA starch showed high levels of coalescence after 3 days and were thus not further analysed. This is to be expected, as the control waxy maize starch (unmodified) is hydrophilic and therefore should not adsorb at oil/water interfaces (Shogren, Viswanathan, Felker, & Gross, 2000). Although it was interesting to note that short term emulsion stability was observed here, this has previously been reported (Li, Li, Sun, & Yang, 2013). At 1% and 1.5% OSA modification, emulsions were more stable and retained their characteristic microstructure for at least 30 days, but with extended storage (90 days) the average droplet size increased and no double emulsions droplets were observed after 90 days. Emulsions stabilised with 2, 2.5 and 3% OSA starch retained their microstructure and were stable for at least 90 days. Droplet diameter remained constant in the case of 2.5 and 3% OSA starch stabilised emulsions. At 2% OSA modification, a slow steady increase in droplet size over storage was noted (presumed to be due to coalescence) although the absolute increase after 90 days was only ∼4 μm.

Emulsion surface topography varied with increasing levels of OSA treatment. Fig. 3 shows cryo-SEM images of representative oil droplets for each of the complex emulsions. The emulsion systems containing unmodified waxy maize starch had a smooth surface and with increasing levels of OSA modification the surface of the emulsions appeared increasingly rough. The formation of a rough emulsion surface was also observed when cocoa particles were used to stabilise o/w emulsions (Gould, Vieira, & Wolf, 2013), suggesting the starch is coating the emulsion surface, and that the enhanced emulsion stability may be partially conferred through a stearic stabilisation mechanism.

### 3.3 Impact of OSA modification level (0–3%) on interfacial digestion and total sodium availability

**Fig. 4:**
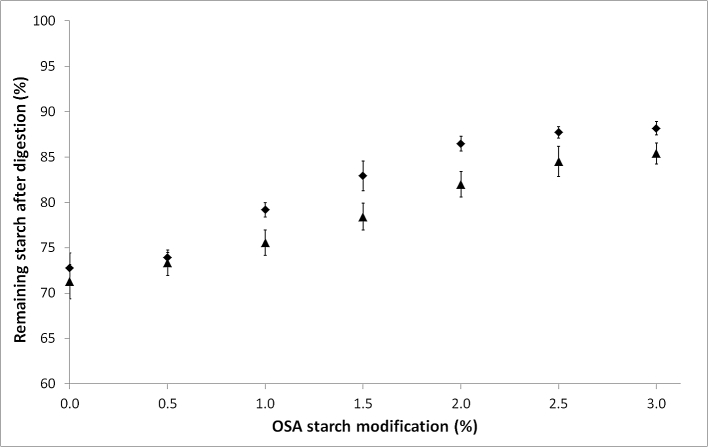
Remaining starch in emulsions stabilised with 0–3% OSA-starch after in vitro (◆) and in vivo (▲) digestion. Data are means of three replicates ± standard deviation.

The starch stabilised w_1_/o/w_2_ emulsions were exposed to α-amylase both in an in vitro assay and in vivo by oral processing, the percentage of non-digested starch was then quantified. The percentage of remaining starch after enzyme digestion increased with increasing level of OSA modification (Fig. 4). OSA modification has previously been reported to affect the rate of bulk starch digestion by rendering bulk starch more resistant to digestion (Han & BeMiller, 2007). As enzyme hydrolysis is initiated from the exterior of the starch (Meireles, Carneiro, DaMatta, Samuels, & Silva, 2009), the presence of OSA groups on the surface reduces enzyme hydrolysis acting as a non-competitive inhibitor to α-amylase due to physical stearic hindrance and increased surface hydrophobicity (He, Liu, & Zhang, 2008). Starch digestion was greatest in vivo and a higher proportion of undigested starch remained in the in vitro emulsion, this may be due to the higher enzyme expression levels of some individuals in vivo compared to the in vitro digestion (50 units/mL), which is within human variability but is in the lower quartile. The variability of the remaining starch values measured was greater in vivo than in vitro, this is suggested to be due to individual variation in salivary flow rate and amylase activity, human salivary α-amylase activity has been shown to vary between individuals between 50 and 400 units/mL (KivelÄ et al., 1997, Mandel et al., 2010).

**Fig. 5:**
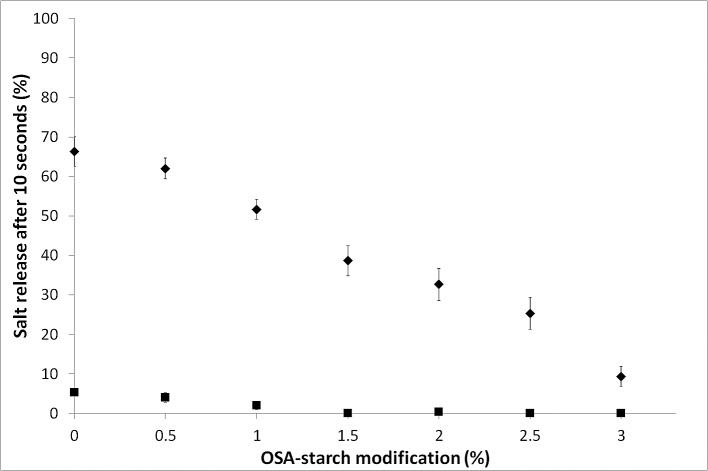
Total salt release after 10 s from emulsions stabilised with different degrees of modified OSA-starch in presence of porcine α-amylase (◆) and in absence of α-amylase (■). Data are means of three replicates ± standard deviation.

In a model in vitro test cell, the complex emulsions were digested using α-amylase and sodium release was tracked using a sodium specific electrode (Fig. 5). Whilst sodium diffused from the internal encapsulated aqueous phase to the continuous phase in all emulsions, this was greatest for the least stable unmodified starch emulsions due to the coalescence of the complex emulsions; high levels of starch modification resulted in a reduced sodium release after 10s, this supports the data shown previously that high levels of OSA treatment increased the stability of the starch’s functionality to enzyme digestion and suggests that an optimum level of OSA modification may offer both physical emulsion stability whilst retaining the ability to digest rapidly and release sodium when exposed to α-amylase.

### 3.4 Sensory perception of varying levels of OSA modified starch stabilised emulsions

#### 3.4.1 Impact of OSA modification level (0–3%) on saltiness perception

**Fig. 6:**
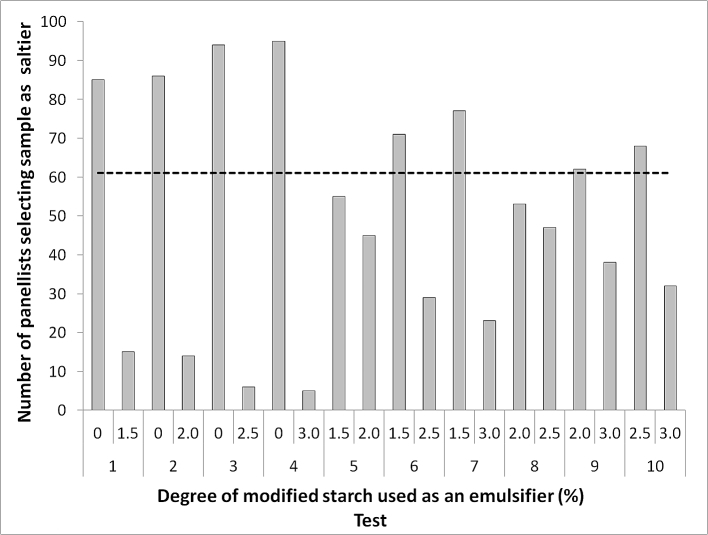
Sample pairs presented in the paired comparison test of emulsions stabilised with five different degrees of OSA-starch modification. The dotted line indicates the minimum number of consensual responses required to conclude significant difference (α = 0.05).

OSA treatment level had a significant (P < 0.05) impact on the overall perception of saltiness by naïve assessors. Fig. 6 shows the results of paired comparison (PC) tests evaluating saltiness intensity of emulsions by naïve assessors. For each PC, the total number of assessors choosing each sample as saltiest is indicated. All tests, with the exception of test 5 and 8, showed a significant difference in saltiness intensity between the samples (this is illustrated as the number of assessor selecting a product exceeding the critical limit shown as a dashed line). In general, results indicated that increasing the degree of starch modification decreased the intensity of saltiness, and the greater the difference in the degree of modification between the two samples, the more likely there was to be measured difference in saltiness. The sample with 0% modification was significantly saltier when compared to any OSA modified sample (tests 1–4) and samples with 1.5% OSA modification were significantly saltier than samples containing starch modified by 2.5% and 3.0% OSA, 1.5% OSA samples did not significantly differ from the 2.0% modification sample. Sample 2.0% was significantly saltier than 3.0% but did not significantly differ from 2.5%. The saltiness perception supports the instrumental measures of salt release recorded in Fig. 5, suggesting that the differences in the level of salt released within the in vitro assay corresponds to the ability of the assessors to discriminate between levels of perceived saltiness.

#### 3.4.2 Impact of OSA modification level (0–3%) on the temporal perception of saltiness

Time-intensity curves of saltiness perception over the time course of consumption of emulsions were obtained from the 10 panellists. Table 1 displays data obtained from three of the extracted parameters; maximum saltiness intensity (*I*_max_), time to reach *I*_max_ (*T*_max_) and the area under the saltiness curve.

*I*_max_ varied significantly among samples (p < 0.01). In general, increasing as the degree of modified OSA starch decreased. The sample with no (0%) OSA modification had the highest *I*_max_ and the sample containing starch with 3.0% OSA modification had the lowest *I*_max_. These results are in agreement with the results of the in vitro study with porcine α-amylase (Fig. 5), where the level of sodium in the continuous phase was higher in emulsions stabilised with lower degrees of modification and demonstrated that the extent of starch modification indeed had an effect on sodium release and affected the intensity of saltiness perception.

The time to maximum intensity (*T*_max_) also varied significantly among the treatment levels (p < 0.01). In general, *T*_max_ of saltiness was reached prior to swallowing at 10 s, with the exception of the emulsion with 3.0% OSA starch modification where maximum intensity occurred at 11 s. *T*_max_ varied depending on the degree of starch with the emulsion sample stabilised with 0% OSA modification having the fastest release (lowest *T*_max_).

The total area under the curve reflects the cumulative saltiness perceived during each test. Total area was greatest for the unmodified samples and the samples with a low level of OSA modification (1.0% and 1.5%), suggesting that total rated sodium was lowest for the more modified samples, this further contributes towards the justification for an optimum OSA modification level.

**Fig. 7:**
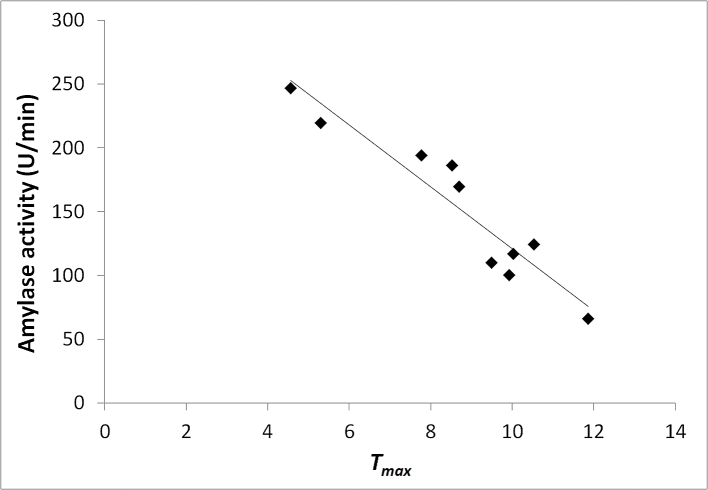
Time to maximum saltiness intensity (*T*_max_) and rate of amylase expression of individual panellist. (R^2^ = 0.8947).

It was also surprising to note that *T*_max_ varied among the panellists (Fig. 7). As the release of salt from the internal phase of the emulsion to the continuous phase for perception is influenced by the presence of α-amylase, salivary flow rates and the amylase activity of panellists it was therefore important to take this into consideration and salivary amylase activity was quantified in each panellist. Salivary α-amylase activities were highly correlated with *T*_max_ values (ρ = −0.95, p < 0.001), panellists with the highest levels of α-amylase activity indicated they reached maximum perceived saltiness much faster compared to those with lower levels of in-mouth enzyme activity. It was interesting to note that the time to maximum saltiness perception varied from ∼12s for the assessors with the lowest salivary α-amylase expression levels to ∼4 s for the assessors with the greatest salivary α-amylase expression levels, this may have a significant impact on taste perception not only in the product studied herein, but also in other food systems.

## 4 Conclusions

This work has validated previous findings that transiently stable complex emulsions, stabilised by OSA starch, can be used to entrap sodium and release in a controlled fashion during oral processing, the work furthered this and identified an optimum level of OSA treatment that confers both emulsion stability whilst delivering sodium orally and resulting in a high level of sodium perception. Whilst these boundaries of success are arbitrary it does serve to suggest that 1.5% OSA treatment may be optimal, and that this could be increased to 2.0% for samples requiring higher levels of inherent emulsion stability. We also observed that individual variation in α-amylase expression resulted in a significant variation in the time to maximum saltiness perception for the complex emulsions, most interestingly this varied from 12 s to 4 s for the assessors with the greatest salivary α-amylase expression levels, highlighting the importance of individual variation on taste perception. This is of relevant to food industry professionals using OSA starch as selection of an intermediate OSA modification level may mitigate against requirements for higher salt levels for consumer liking, especially in product where complex w/o/w emulsions are formed either by design or as an artefact of processing or mastication.

## Figures and Tables

**Table 1:** ANOVA of maximum saltiness intensity (*I*_max_), time to maximum saltiness intensity (*T*_max_) and area under the curve for six different emulsions stabilised with different degree of modified OSA starch, with salt encapsulated in the internal phase. Samples with the same letter code in a row are not significantly different (*p* > 0.05).

	OSA modification (%)						
	0	0.5	1.0	1.5	2.0	2.5	3.0
*T*_max_*(s)*	4.12^a^	5.35^a^	8.72^b^	8.64^b^	9.98^bc^	9.42^bc^	11.16^c^
*I*_max_	9.262^d^	9.231^d^	9.012^cd^	9.116^cd^	8.488^ab^	8.206^b^	7.196^a^
Area under the curve	240.2^d^	238.7^d^	223.3^cd^	225.9^cd^	204.9^ab^	194.2^b^	162.3^a^
